# Emerging Dietary Bioactives in Health and Disease

**DOI:** 10.3390/nu15081956

**Published:** 2023-04-19

**Authors:** Elad Tako

**Affiliations:** Department of Food Science, Cornell University, Stocking Hall, Ithaca, NY 14853-7201, USA; et79@cornell.edu

This monograph, based on a Special Issue of Nutrients, contains 16 manuscripts—2 review manuscripts and 14 original research manuscripts—that reflect the wide spectrum of currently conducted research in the field of Emerging Dietary Bioactives in Health and Disease. The manuscripts in this Special Issue collection include contributions from researchers from multiple countries, including the USA, Brazil, United Kingdom, Italy, Norway, China, and Israel. The presented manuscripts cover a wide variety and range of topics in the field of dietary bioactives in health and disease, with emphasis on the investigation of the effects hydrolyzed protein of chia (*Salvia hispanica* L.) and *Lacticaseibacillus paracasei* on intestinal functionality, morphology, and bacterial populations, in vivo (*Gallus gallus*) [1]; the demonstration of the capacity of macauba (*Acrocomia aculeata*) pulp oil to prevent adipogenesis, inflammation and oxidative stress in mice fed a high-fat diet [2]; the demonstration of the capacity of kombuchas from green and black tea to modulate the gut microbiota and improve the intestinal health of wistar rats fed a high-fat high-fructose diet [3]; the methodological preparation, characterization, wound healing, and cytotoxicity assay of pegylated nanophytosomes loaded with 6-gingerol [4]; the discussion of empire apple (*Malus domestica*) juice, pomace, and pulp and the modulation of intestinal functionality, morphology, and bacterial populations in vivo (*Gallus gallus*) [5]; the effect of chia (*Salvia hispanica* L.) associated with high-fat diet on the intestinal health of wistar rats [6]; the demonstration of the capacity of curcumin-added whey protein to positively modulate skeletal muscle inflammation and oxidative damage after exhaustive exercise [7]; the introduction of the novel intra-amniotic administration—an emerging method with which to investigate necrotizing enterocolitis in vivo (*Gallus gallus*) [8]; the investigation of the effect of black corn anthocyanin-rich extract (*Zea mays* L.) on cecal microbial populations in vivo (*Gallus gallus*) [9]; the demonstration of BRD9 inhibition by natural polyphenols which target DNA damage/repair and apoptosis in human colon cancer cells [10]; the alterations in intestinal brush border membrane functionality and bacterial populations following the intra-amniotic administration (*Gallus gallus*) of catechin and its derivatives [11]; the performance of a comparison of the effects of concord grape (*Vitis labrusca* L.) puree, juice, and pomace on intestinal morphology and functionality, and on bacterial populations in vivo (*Gallus gallus*) [12]; the demonstration of how the intra-amniotic administration (*Gallus gallus*) of genistein alters mineral transport, intestinal morphology, and gut microbiota [13]; the occurrence of the alterations in intestinal brush border membrane functionality and bacterial populations following intra-amniotic administration (*Gallus gallus*) of nicotinamide riboside and its derivatives [14]; a literature review and discussion of dietary trehalose as a bioactive nutrient [15]; and an analysis of how resistant maltodextrin consumption in a double-blind, randomized, crossover clinical trial induces specific changes in potentially beneficial gut bacteria [16]. These wide spectra of topics further demonstrate the importance and relevance of dietary bioactive compounds, as well as their relevance in health and disease circumstances.

Plant-based diets contain wide varieties of metabolites that may impact on health and disease prevention. Most are focused on the potential bioactivity and nutritional relevance of several classes of phytochemicals, such as polyphenols, flavonoids, carotenoids, phyto-oestrogens, and frucrooligo-saccharides [17]. These compounds are found in fruit, vegetables, and herbs [18]. The permitted daily intakes of some of these compounds may exceed 100 mg. Moreover, intestinal bacterial activity may transform complex compounds such as anthocyanins, procyanidins, and isoflavones into simple phenolic metabolites [19]. The colon is thus a rich source of potentially active phenolic acids that may impact both locally and systemically on gut health. Further, nondigestible fibers (prebiotics) are dietary substrates that selectively promote proliferation and/or activity of health-promoting bacterial populations in the colon [20]. Prebiotics, such as inulin, raffinose, and stachyose, have a proven ability to promote the abundance of intestinal bacterial populations, which may provide additional health benefits to the host [21,22,23,24,25,26]. Further, various pulse seed soluble (fiber) extracts are responsible for improving gastrointestinal motility, intestinal functionality and morphology, and mineral absorption [25,27]. Studies have indicated that the consumption of seed origin soluble extracts can upregulate the expression of BBM proteins that contribute to the digestion and absorption of nutrients. Soluble extracts can positively affect intestinal health by increasing the mucus production, goblet cells number/diameter, villus surface area, and crypt depth [25,27]. These functional and morphological effects appear to occur due to the increased motility of the digestive tract, leading to hyperplasia and/or hypertrophy of muscle cells. Plant-origin soluble extracts may act, directly or indirectly, to increase mineral solubility and, therefore, dietary bioavailability. This occurs due to fiber fermentation and the bacterial production of SCFA, a process that reduces intestinal pH, inhibits the growth of potentially pathogenic bacterial population and increases the solubility and, therefore, absorption of minerals. The SCFA can increase the proliferation of epithelial cells, which, in turn, increases the absorptive surface area, an occurrence which contributes to the absorption of nutrients [28,29,30]. Several phenolic acids and other phytochemicals affect the expression and activity of enzymes involved in the production of inflammatory mediators of pathways thought to be important in the development of gut disorders, including colon cancer. However, it is still unclear as to which of these compounds are beneficial to gut health. Hence, the aim of the current Special Issue is to further explore the interactions between dietary bioactive compounds and overall health, and to further expand and add research knowledge on the vital role of dietary bioactive compounds in various nutrition-related physiological and metabolic pathways, and beyond.

This Special Issue and collection of manuscripts constitutes a useful summary of progress in various areas related to emerging dietary bioactives in health and disease.
